# A polyethersulfone–bisphenol sulfuric acid hollow fiber ultrafiltration membrane fabricated by a reverse thermally induced phase separation process

**DOI:** 10.1039/c7ra12602f

**Published:** 2018-02-19

**Authors:** Sheng-Hui Liu, Min Liu, Zhen-Liang Xu, Yong-Ming Wei

**Affiliations:** State Key Laboratory of Chemical Engineering, Membrane Science and Engineering R&D Lab, Chemical Engineering Research Center, East China University of Science and Technology (ECUST) 130 Meilong Road Shanghai 200237 China; Shanghai Key Laboratory of Advanced Polymeric Materials, Key Laboratory for Ultrafine Materials of Ministry of Education, School of Materials Science and Engineering, ECUST 130 Meilong Road Shanghai 200237 China liumin@ecust.edu.cn chemxuzl@ecust.edu.cn +86-21-64252989 +86-21-64253670

## Abstract

A novel antifouling polyethersulfone (PES) hollow fiber membrane was modified by the addition of bisphenol sulfuric acid (BPA-PS) using a reverse thermally induced phase separation (RTIPS) process. BPA-PS was synthesized by click chemistry and was blended to improve the hydrophilicity of PES hollow fiber membranes. The performance of PES/BPA-PS hollow fiber membranes, prepared with different contents of BPA-PS and at different temperatures of the coagulation water bath, was characterized by scanning electron microscopy (SEM), pure water flux (*J*_w_), BSA rejection rate (*R*), atomic force microscopy (AFM), X-ray photoelectron spectroscopy (XPS), Fourier transform infrared spectroscopy (FTIR) and water contact angle measurements. SEM morphologies revealed that a finger-like cross-section emerged in the hollow fiber membrane by a non-solvent induced phase separation (NIPS) mechanism while a sponge-like cross-section appeared in the hollow fiber membrane *via* the RTIPS method. Both FTIR and XPS analysis indicated that the sulfate group in BPA-PS was successfully blended with the PES membranes. The results from AFM and water contact angle measurements showed that the surface roughness increased and the hydrophilicity of the PES/BPA-PS hollow fiber membrane was improved with the addition of BPA-PS. The results demonstrated that the PES/BPA-PS membrane with 1 wt% BPA-PS *via* RTIPS exhibited optimal properties.

## Abstract 2

A novel antifouling polyethersulfone (PES) hollow fiber membrane was modified by the addition of bisphenol sulfuric acid (BPA-PS) using a reverse thermally induced phase separation (RTIPS) process.

## Graphical abstract

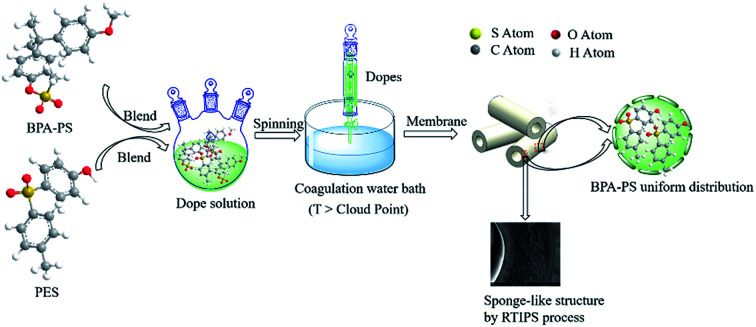

## 1 Introduction

Owing to its advantages of moderate operation conditions, less energy consumption and environmental friendly properties, membrane separation techniques^1–4^ have become the most popular approach in water purification. Among a variety of membrane preparation methods, phase separation methods, which include the non-solvent induced phase separation method (NIPS)^5–8^ and thermally induced phase separation (TIPS) method,^9–12^ are widely used for membrane preparation with outstanding merits. During the NIPS process, the fast transfer of solvent and non-solvent leads to the phase separation, which is beneficial for the formation of a dense skin top surface, finger-like structure and poor mechanical properties.^13–15^ In the TIPS process, a homogeneous doping solution is prepared at high temperature and spun at room temperature.^16,17^ Nevertheless, the application of the TIPS procedure is limited by its requirement for high temperature and fewer options of appropriate diluents.

As a novel method to control phase separation, reverse thermally induced phase separation (RTIPS), which is the lower critical solution temperature (LCST)-TIPS process,^18^ combines advantages of both low temperature in dope solutions preparation (similar to NIPS) and the fast rate of heat transfer (similar to TIPS). Compared to NIPS method, the structure of the membrane formed from RTIPS transforms the finger-like structure into sponge-like structure. In the meantime, the dense skin layer turns into the porous layer. The sponge-like structure accelerates the rate of BSA rejection and provides excellent mechanical properties, while porous surface contributes to high pure water flux. Contrast with TIPS, dopes are obtained at room temperature and spun at a temperature higher than cloud point, which could reduce the energy consumption and makes it more conducive to industrialization. In a word, RTIPS process is a cost-effective process and generates less waste. However, due to the membrane is more vulnerable to pollutants, the popularization and development of the membrane technology is severely hampered. In order to improve the antifouling property of the membrane, the modification is necessary.^19^ Zhao *et al.*^20^ added HBPE into PSF membrane *via* RTIPS method, then both the antifouling property and water permeate rate were improved to a great extent. Liu *et al.*^21^ prepared PES/TiO_2_ hybrid membrane through sol–gel process by the assistant of RTIPS method. Thus, the antifouling property of the membrane was enhanced with the addition of TiO_2_.

PES hollow fiber membranes need to be fabricated to achieve excellent properties. Among the modified methods, blending approach is a simple and useful route to prepared PES membrane. Daraei *et al.*^22^ mixed various types of polymers to modify multi-walled carbon nanotubes to improve the antifouling capability of PES membrane. Li *et al.*^23^ added SPES into PES hollow fiber membrane to improve the anti-pollution properties. However, the process and mechanism are more complex to form a membrane in these approaches. Click chemistry offers a terrific way for membrane modification.^24–28^ The prominent merits of click chemistry are its capacity to supply superior site selectivity, which can provide almost quantitative conversion under mild condition and with almost no side reaction or by-product.^29–31^ Ge *et al.*^32^ constructed an ionic highway in the anion-exchange membrane by click chemistry and synthesized a novel AEMs material. Zheng *et al.*^33^ developed an antifouling silicon surface with improving the hemocompatibility by click chemistry, and found that the antifouling property enhanced in an artificial kidney equipment. Besides, the modification of polyethersulfone membrane with BPA-PS by RTIPS method has not been reported in the literature. Thus, a novel approach was proposed to introduce the click sulfuric acid functional group into polyethersulfone membrane. It is very interesting to point out that the click sulfuric acid functional group in bisphenol A poly sulfuric acid (BPA-PS)^34^ may facilitate the antifouling performance in the polyethersulfone membrane. As a new antifouling material, BPA-PS (synthesized by click reaction) not only displays excellent antifouling property against protein fouling but also provides an effective approach to modify the polyethersulfone membrane.

To investigate the polyethersulfone membrane modified by BPA-PS further, the effects of coagulation water bath temperature and the quantity of BPA-PS on membrane formation mechanism, membrane structure, membrane filtration performance as well as fouling resistance were investigated in this work.

## 2 Experimental

### 2.1 Materials

Polyethersulfone (PES, *M*_w_ = 45 000) was purchased from BASF Co. Ltd. (German). PES was used for membrane preparation after dried about 24 h at 333 K. *N*,*N*-Dimethyl acetamide (DMAC), diethylene glycol (DEG) were provided by Shanghai Chemical Reagent Co. Ltd. Bisphenol sulfuric acid (BPA-PS, *M*_w_ = 97 000) was provided by Shanghai Institute of Organic Chemistry, Chinese Academy of Sciences. Bovine serum albumin (BSA, *M*_w_ = 67 000) was provided by Shanghai Lianguan Biochemical Engineering Co. Ltd. All of the pure water in this work was self-made.

### 2.2 Characterization of BPA-PS

The chemical structure of BPA-PS was characterized by FTIR (Nicolet 6700, Thermo Electron Scientific Instruments Corp.).

### 2.3 Preparation of dope solutions

The solvent (DMAc) and a certain amount of BPA-PS were premixed firstly in a conical flask about several hours, then measured amount of PES was dissolved in the conical flask at room temperature (about 293 K), finally, non-solvent (DEG) was added in the solution. The dope solutions degassed about 12 h at room temperature under atmospheric pressure for spinning. The composition of the dopes and the coagulation water bath temperatures were listed in Table 1 and 2, respectively.

**Table 1:** The compositions of dopes

Dope no	Composition of dope solution (wt%)			
	PES	BPA-PS	DMAc	DEG
MBPA PS-1	17.00	0.00	43.00	40.00
MBPA PS-2	17.00	0.50	42.75	39.75
MBPA PS-3	17.00	1.00	42.48	39.52
MBPA PS-4	17.00	1.50	42.28	39.27
MBPA PS-5	17.00	2.00	41.97	39.03

**Table 2:** Coagulation water bath temperature

Membrane no	Water bath temperature (K)	Membrane no	Water bath temperature (K)
MBPA PS-1-50	323	MBPA PS-4-50	323
MBPA PS-2-50	323	MBPA PS-5-50	323
MBPA PS-3-50	323	MBPS PS-3-20	293

### 2.4 Preparation of hollow fiber membrane

**Fig. 1:**
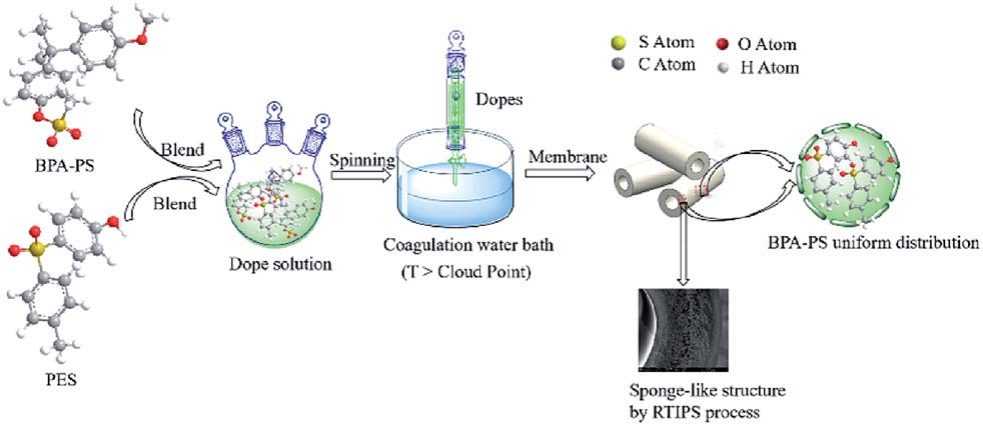
Preparation illustration of the PES/BPA-PS membrane.

The preparation process of the PES hybrid hollow fiber membrane by wet-spinning method was illustrated in Fig. 1. Both the internal and external coagulation water bath were deionized water, the temperatures of the coagulation water bath were listed in Table 2. The dope solution flow rate, bore fluid rate and spinning flow rate were constant. These prepared membranes were immersed in deionized water about 3 d which were refreshed every day to remove the remnant solvent in the hollow fiber membranes, then the hollow fiber membranes were immersed in glycerol aqueous solution 3 d and were dried in air at room temperature for testing.

### 2.5 Measurement of light transmittance

The experiment of light transmittance was operated *via* a self-made device.^35^ A collimated laser was directed into a glass sheet which was immersed in water bath rapidly at fixed temperature. The message of light intensity was captured *via* a light detector and was recorded in computer. The precipitation rate of dopes was characterized by the curve of light transmittance to immersion time.

### 2.6 Cloud point measurement

The cloud point measurement was performed in order to confirm the phase separation temperature (*i.e.*, LCST, which was defined as cloud point) of dopes.^18^ The cloud points of dopes were measured *via* a self-made device. Briefly, the dope solution was inserted between two cover slips which were heated on a hot stage (KEL-XMT-3100, Shanghai Weitu Optics and Electron Technology Co. Ltd.) from 293 K to 373 K at 1 K min^−1^. A collimated laser was directed on coverslips, then the light intensity, which was captured by a light detector, was recorded on the computer. The intensity of transmitted light decreased rapidly with the phase separation occurred. The change of the signal was considered as the indication of cloud point.

### 2.7 SEM measurement

Morphologies (top surface and cross section) of hollow fiber membranes were visualized *via* a scanning electron microscope (SEM; Nova NanoSEM, USA). The cross-sections of the membranes were broken in liquid nitrogen. Each sample was coated with gold in vacuum environment.

### 2.8 Permeation measurement

The pure water flux (*J*_w_) and BSA rejection rate (*R*) of hollow fiber membranes were measured by a self-made device.^36^ All tests were executed at room temperature along with a successive pressure (0.1 MPa). The *J*_w_, *R* of the hollow fiber membranes, which were evaluated precisely using deionized water and BSA aqueous solution (300 mg L^−1^), were calculated based on the following eqn (1) and (2), respectively.1
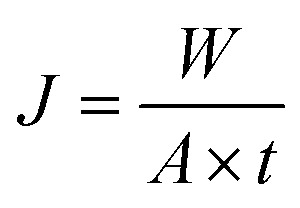
2
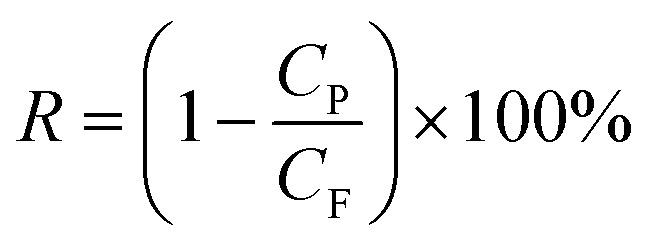
where *J*_w_ is the pure water flux (L m^−2^ h^−1^), *A* is the effective membrane area (m^2^), *Q* is the permeate volume (L), *t* is the permeate time (h), *R*, *C*_p_ and *C*_F_ were the BSA rejection rate (%), permeate and feed solutions (wt%), respectively.

### 2.9 Porosity and size measurement

The membrane porosity *ε* (%) was evaluated by gravimetric method using the following equation:3
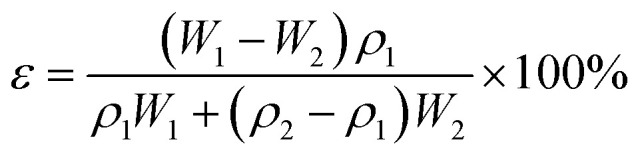
where *ε*, *W*_1_, *W*_2_, *ρ*_1_ and *ρ*_2_ are the porosity of the membrane, wet membranes weight, dry membranes weight, water density and the polymer (PES) density, respectively.

Mean pore radius *r*_m_ (μm) of the membrane was defined according to the formula of Guerout–Elford–Ferry:^36^4
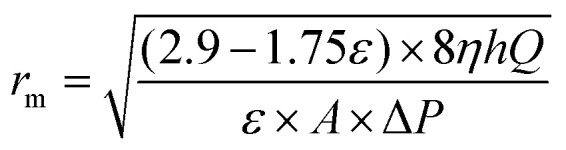
where *η* is the viscosity of water (8.9 × 10^−4^ Pa s^−1^), *h* is the thickness of the membrane (mm), Δ*P* is the operate pressure (0.1 MPa).

Maximum pore size *r*_max_ (μm) of the membrane could be obtained through bubble point procedure referring to the Laplace's equation:^37^5
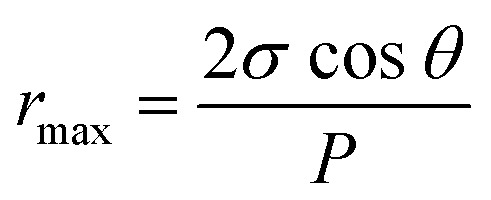
where *σ*, *θ*, *P* are the water surface tension (22.8 × 10^−3^ N m^−1^), the contact angle (°) of the membrane and the minimum bubble point pressure (MPa), respectively.

### 2.10 Water contact angle measurement

The contact angle (*θ*) was acquired by the device (JC2000A, Shanghai Zhongcheng Digital Equipment Co. Ltd., China) at room temperature. Briefly, the camera capturing image obtained when droplet fell on the surface of the membrane as well as sample contact angle was recorded in computer. Every sample was measured four times and then averaged.

### 2.11 AFM measurement

Atomic force microscopy (Veeco, NanoscopeIIIa Multimode AFM) with 5 mm × 5 mm scanning size was employed to examine the roughness of the membrane. The AFM figure was managed by Gwyddion software, moreover, the roughness of the membrane was analysed by NanoScope analysis software.

### 2.12 XPS measurement

X-ray photoelectron spectroscopy analysis (XPS; VG Microlab II, UK) was used for determining chemical composition of the membrane.

### 2.13 Antifouling measurement

A dead end filtration experiment was performed to examine the long-term antifouling property of the membrane further.^38^ Briefly, membrane pure water flux (*J*_w1_, L m^−2^ h^−1^) was obtained at 0.1 MPa. Afterwards, BSA solution (300 mg L^−1^) was permeated through the tested membrane to achieve the BSA flux (*J*_p1_, L m^−2^ h^−1^) followed by washing the tested membrane by chemical process. The experiment repeated twice. The FRR (%) of the membrane, which obtained according to the permeation fluxes, was defined by the following formula (6). The pure water flux and BSA flux with time also obtained to illustrate the anti-pollution properties of the membranes further.6
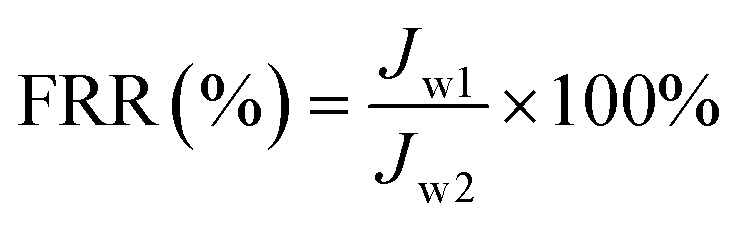


## 3 Results and discussion

### 3.1 Characterization of BPA-PS

**Fig. 2:**
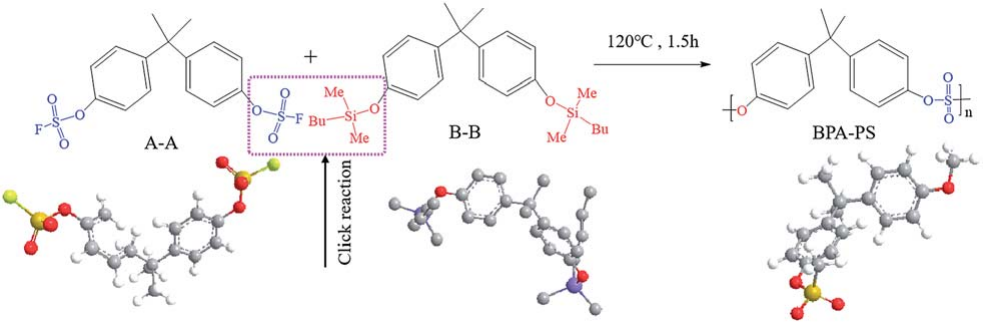
The synthetic route of BPA-PS by click chemistry.

**Fig. 3:**
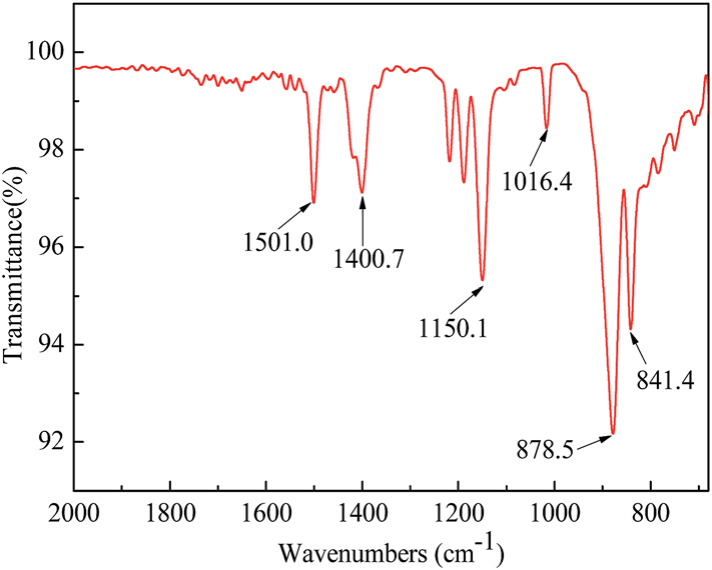
The FTIR spectra of BPA-PS.

The synthetic pathway and the FTIR spectrum of BPA-PS were displayed in Fig. 2 and 3, respectively. In Fig. 2, the click reaction occurred between the –SO_3_F and –OTBS at 120 °C about 1.5 h, then the –SO_4_– generated. As shown in Fig. 3, the absorption peak at 1150.1 cm^−1^ represented the symmetrical stretching vibrations of the S=O in the sulfuric acid group. In addition, the absorption peak at 878.5 cm^−1^ represented the stretching vibrations of the C–O–S in the sulfuric acid group. These results indicated that BPA-PS molecules had a high hydrophilicity group.

### 3.2 Cloud point

For the LCST dope solution which composed of PES/BPA-PS/DMAc/DEG, phase separation occurred when the temperature of the dope solution increased to the cloud point.

### 3.3 Cloud point

For the LCST dope solution which composed of PES/BPA-PS/DMAc/DEG, phase separation occurred when the temperature of the dope solution increased to the cloud point. The dope solution was thermodynamically stable at room temperature, while the interaction between the mixed polymer and solvent became increasingly weak with the increased coagulation bath temperature. Thus, the phases separated when the temperature reached cloud point. The influence of the BPA-PS from 0 wt% to 2 wt% on the cloud point was not obvious, and the cloud point was about 313 K.

### 3.4 Viscosity and light transmittance

**Fig. 4:**
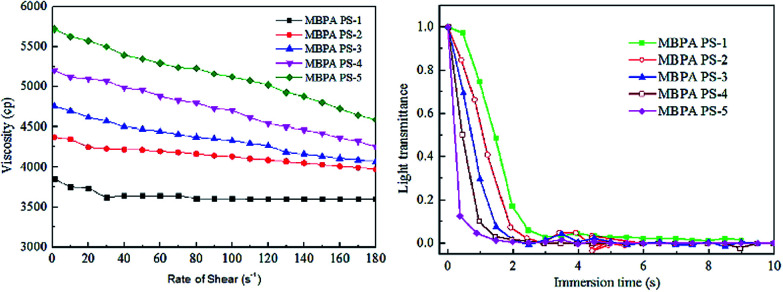
The viscosity and light transmittance curves of the dopes with BPA-PS.

Fig. 4 revealed the rate of light transmittance and the viscosity of the dope solutions. The viscosity in Fig. 4 indicated that the viscosity increased and exhibited a shear-thinning phenomenon in the presence of BPA-PS. This phenomenon became more pronounced when the mass ratio of BPA-PS increased from 0 wt% to 2 wt%. Moreover, Fig. 4 illustrated that the transmittance curve descended speedily at the initial stage and was accelerated with the addition of BPA-PS. In general, high viscosity^39^ would decrease the precipitated speed, however, the rate of phase separation was accelerated by the increased viscosity. This phenomenon could be explained through that the membranes formation mechanism was dominated *via* RTIPS process when the coagulation bath temperature was higher than the cloud point, and the speed of heat transfer was much higher than the mass transfer under the RTIPS mechanism. For this reason, the transmittance curve descended speedy at the initial stage and was accelerated with the increased BPA-PS.

### 3.5 XPS analytical result of PES/BPA-PS hollow fiber membrane

**Fig. 5:**
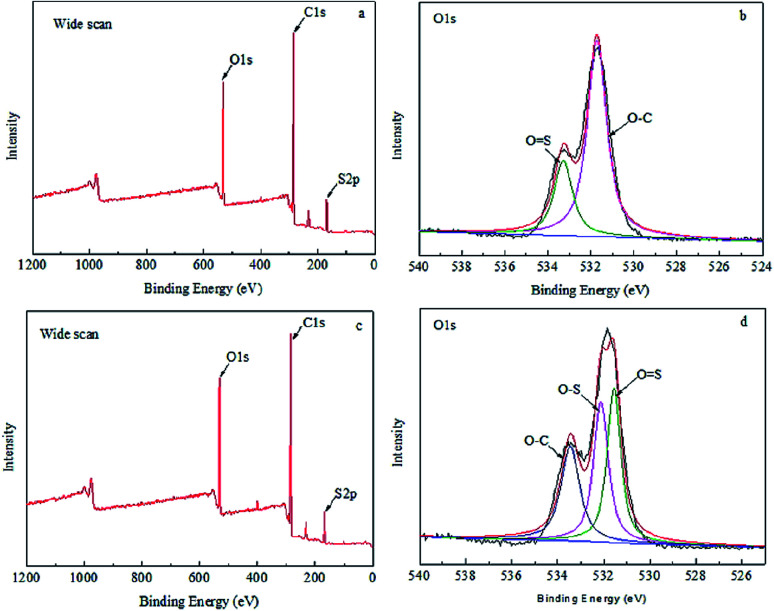
(a and c) Wide scan of MBPA PS-1-50 and MBPA PS-3-50, (b and d) O1s region of MBPA PS-1-50 and MBPA PS-3-50. Coagulation temperature was 323 K.

XPS results of MBPA PS-1-50 and MBPA PS-3-50 were illustrated in Fig. 5 and Table 3. There were three broaden peaks at 172 eV, 296 eV and 536 eV in the blank PES membrane (Fig. 5a) which represented S2p, C1s and O1s regions, respectively. O1 score-level spectrum of the membrane included two peaks at 531.8 eV and 533.3 eV, which corresponding represented O=S and O–C. It was worth noting that a new peak appeared at 532.3 eV, which represented that O–S appeared in the PES/BPA-PS membrane (Fig. 5d). The appearance of the new peak showed that the PES/BPA-PS membranes had already blended with BPA-PS.

**Table 3:** Elementary compositions of PES/BPA-PS hollow fiber membranes

Sample	Atom percent (%)			
	C	O	S	H
MBPA PS-1-50	69.79	21.47	5.71	3.03
MBPA PS-3-50	69.76	22.13	6.30	1.81

### 3.6 Membrane structure of PES/BPA-PS hollow fiber membrane

**Fig. 6:**
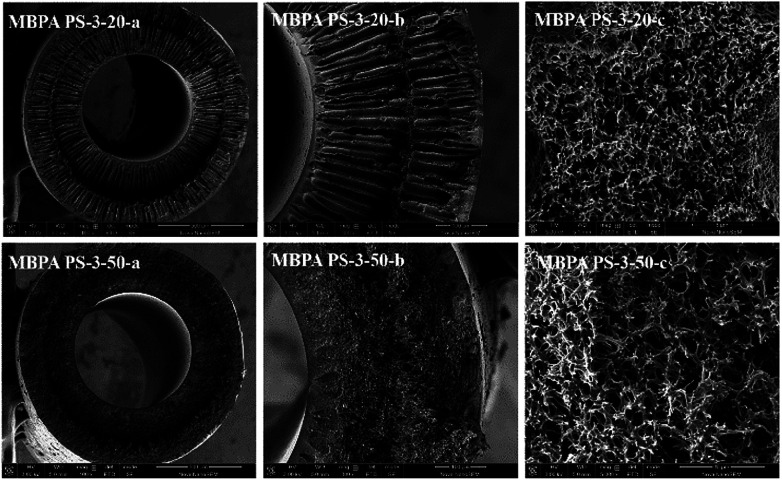
SEM micrographs of MBPA PS-1-20 and MBPA PS-1-50. (a) Full cross-section (magnification 100×); (b) part cross-section (magnification 350×); (c) enlarge cross-section (magnification 10 000×).

**Fig. 7:**
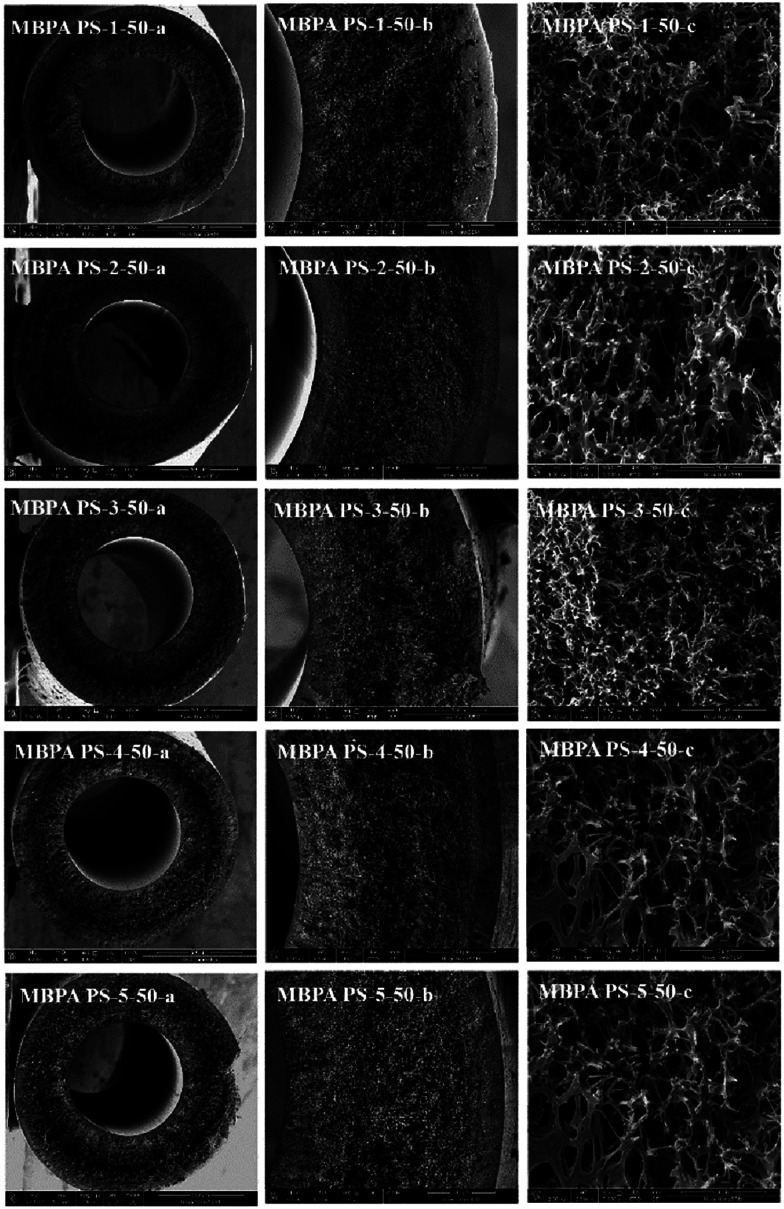
SEM micrographs of the membranes with BPA-PS. Coagulation water bath temperature was 323 K. (a) Full cross-section (magnification 100×); (b) part cross-section (magnification 350×); (c) enlarge cross-section (magnification 10 000×).

SEM images of the membranes were exhibited in Fig. 6 and 7, respectively. The working mechanism of the dopes underwent NIPS process when the coagulation bath temperature was lower than the cloud point while RTIPS took an effective effect when coagulation bath temperature was higher than the cloud point. As illustrated in Fig. 6, the finger-like structure could be found in the MBPA PS-3-20 (by NIPS) while the sponge-like structure was shown in MBPA PS-3-50 (by RTIPS). Owing to the common rule that thermodynamically low stability of dopes and the hydrophilicity of BPA-PS strengthened the precipitating rate by facilitating the inflow rate of pure water, then led to the formation of finger-like structures in MBPA PS-3-20. On the other hand, as it widely known that the speed of heat transfer is much higher than the rate of mass transfer, then the heat transfer is the major driving force in membrane formation procedure. Thus, when the coagulation bath temperature is higher than the cloud point (RTIPS process), the finger-like structure turns into sponge-like structure in MBPA PS-3-50.

As shown in Fig. 7, the cross-sections of the membranes varied with the addition of BPA-PS when coagulation water bath temperature was fixed at 323 K. MBPA PS-1-50 showed the macroporous structure (MBPA PS-1-50-a) and dense cross-section (MBPA PS-1-50 b) under SEM images which indicated the rapid exchange of solvent and non-solvent during the membrane formation procedure. To investigate the influence of BPA-PS on the formation of various morphologies, SEM photographs, as shown in Fig. 7, turned dense cross-section and macroporous structure into sponge-like structure with the addition of BPA-PS. This could be explained by the increased viscosity of BPA-PS, which delayed the speed of phase separation and then sponge-like structure appeared in the membranes. Stratification in the middle of the membrane was ascribed to that the pure water transferred from interior and exterior to middle layer of the membrane simultaneously with fast transfer speed,^40,41^ and then led to pure water accumulated in the middle layer of the membrane. Finally, macroporous structure appeared in the cross-section of the membrane.

### 3.7 Pore size and porosity of PES/BPA-PS hollow fiber membrane

The porosity and pore size of the membranes were listed in Table 4, which varied with the addition of BPA-PS and membrane formation mechanism (NIPS or RTIPS). The porosity of the membranes MBPA PS-1-50, MBPA-PS-2-50, MBPA PS-3-50, MBPA PS-4-50 and MBPA PS-5-50, which prepared by RTIPS method, exhibited an increase first and then decreased trend with the addition of BPA-PS. Moreover, the porosity of the membranes with BPA-PS was higher than that of the blank PES membrane obviously. With regard to the membrane MBPA PS-3-20 and MBPA PS-3-50, the porosity increased when the formation mechanism of the membrane changed from NIPS to RTIPS. The porosity in Table 4 was uniform with the pure water flux and the BSA rejection rate in Fig. 10 and 11.

**Table 4:** Pore size and porosity of PES/BPA-PS membrane

Membrane No	BPA-PS (wt%)	Coagulation bath temperature (K)	Porosity (%)	*r*_max_ (μm)	*r*_m_ (μm)
MBPA PS-1-50	0	323	80.3 ± 0.3	0.40 ± 0.03	0.083 ± 0.003
MBPA PS-2-50	0.5	323	85.6 ± 0.5	0.41 ± 0.01	0.112 ± 0.011
MBPA PS-3-50	1.0	323	86.7 ± 0.1	0.59 ± 0.01	0.109 ± 0.001
MBPA PS-4-50	1.5	323	86.1 ± 0.5	0.56 ± 0.01	0.101 ± 0.004
MBPA PS-5-50	2.0	323	86.3 ± 0.2	0.53 ± 0.01	0.097 ± 0.005
MBPA PS-3-20	1.0	293	85.5 ± 0.2	0.51 ± 0.01	0.093 ± 0.007

As for the MBPA PS-3 series, the maximum pore size and mean pore size of the MBPA PS-3-50 (produced from RTIPS) were higher than the MBPA PS-3-20 (produced from NIPS), and this result matched with the SEM photographs in Fig. 6. For the membranes (MBPA PS-1-50, MBPA-PS-2-50, MBPA PS-3-50, MBPA PS-4-50 and MBPA PS-5-50) prepared *via* RTIPS method, the *r*_max_ and *r*_m_ of the membrane with BPA-PS were higher than that of pure PES membranes due to the hydrophilicity of BPA-PS. For the MBPA-PS-2-50, MBPA PS-3-50, MBPA PS-4-50 and MBPA PS-5-50, the *r*_max_ and *r*_m_ decreased with the increased BPA-PS, which was mainly caused by the high viscosity with the addition of BPA-PS.

### 3.8 AFM and water contact angle analysis of PES/BPA-PS hollow fiber membrane

**Fig. 8:**
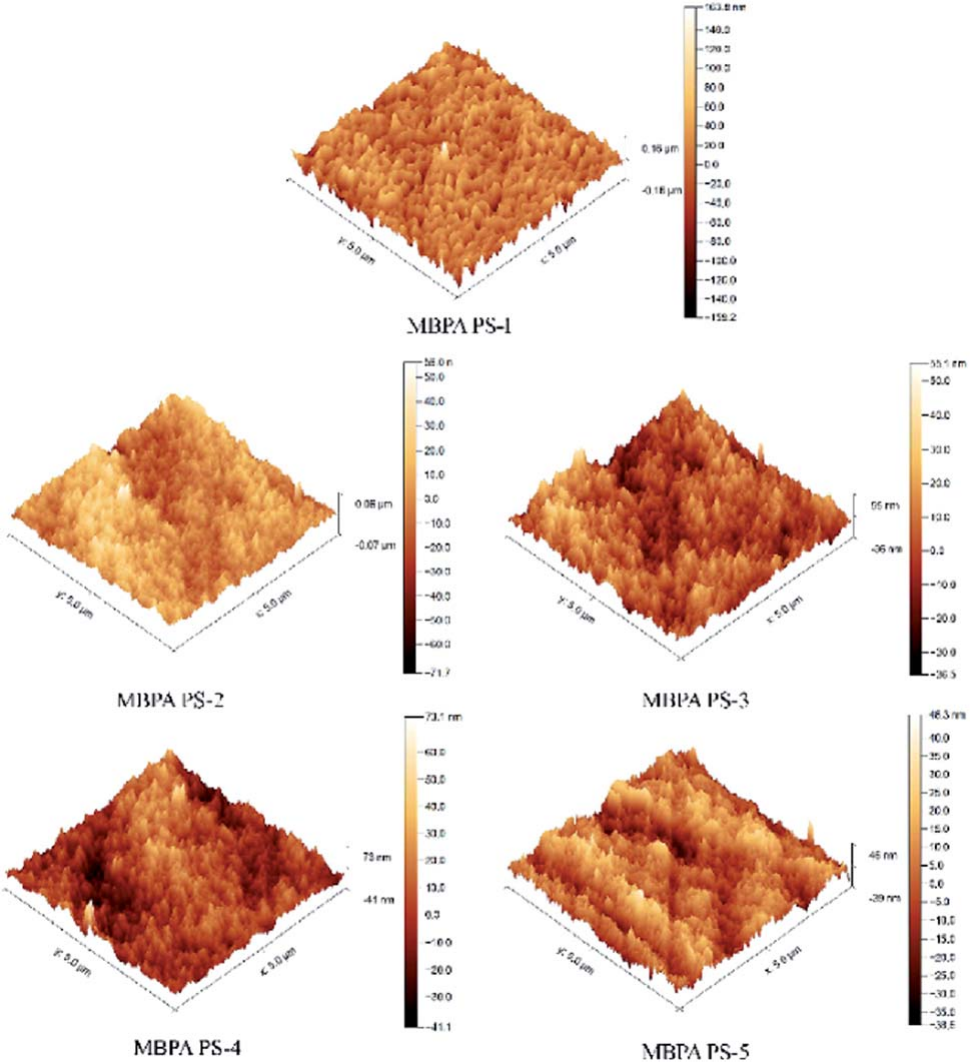
AFM images of PES/BPA-PS hollow fiber membrane. Coagulation water bath temperature was 323 K.

Fig. 8 and Table 5 exhibited the three-dimensional AFM images and the roughness of the membranes. In the scan area 5 μm × 5 μm, the *R*_a_ of MBPA PS-1, MBPA PS-2, MBPA PS-3, MBPA PS-4 and MBPA PS-5 were 10.5 nm, 13.5 nm, 17.3 nm, 22.7 nm and 21.5 nm, respectively. The AFM image of the blank membrane, as illustrated in Fig. 8, was comparatively smooth. While the AFM images of the membrane became coarser with the increased BPA-PS. The anti-fouling property improved with the roughness because the pollutant accumulated in “Valley” in the membrane surface. BPA-PS, which increased the hydrophilicity of the membrane, also enhanced the adsorbance of contaminants on membrane than the blank PES membrane, and then improved the antifouling property of the membrane.

**Table 5:** Roughness of PES/BPA-PS hollow fiber membrane

Membrane no	*R*_a_ (nm)	*R*_ms_ (nm)
MBPA PS-1	10.5	13.2
MBPA PS-2	13.5	16.4
MBPA PS-3	17.3	22.5
MBPA PS-4	22.7	30.2
MBPA PS-5	21.5	29.5

**Fig. 9:**
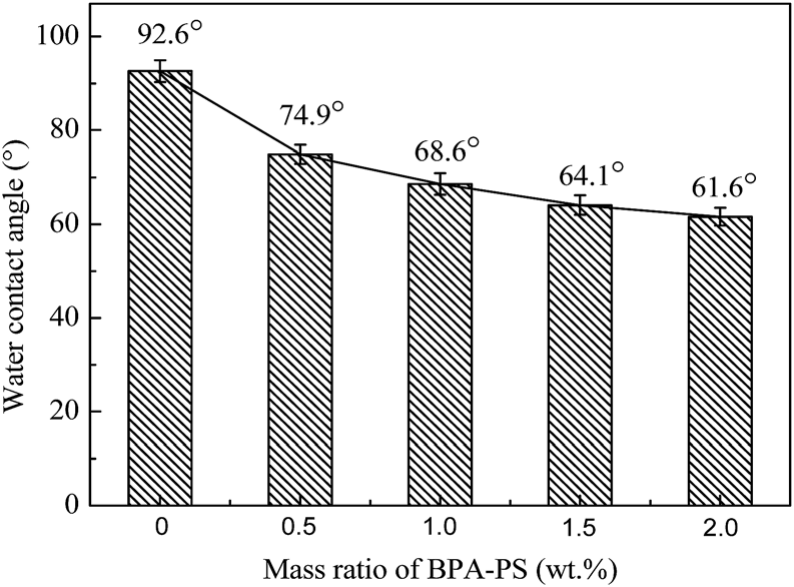
The static water contact angles of PES/BPA-PS hollow fiber membrane. Coagulation temperature was 333 K.

The water contact angle was metered by trailing the top surface of the membrane. As illustrated in Fig. 9, the static water contact angles of MBPA PS-1-50, MBPA PS-2-50, MBPA PS-3-50, MBPA PS-4-50 and MBPA PS-5-50 were 92.6°, 74.9°, 68.6°, 64.1° and 61.6°, respectively, which exhibited a decreasing trend with the increased BPA-PS. The presence of the hydroxyl group in BPA-PS and the transformation of BPA-PS to the top surface could explain the decreasing trend. As can be known that lower water contact angle is benefited for excellent anti-fouling property.^42^ Therefore, the addition of BPA-PS improved the hydrophilicity of the membrane.

### 3.9 Permeation performance of PES/BPA-PS hollow fiber membrane

**Fig. 10:**
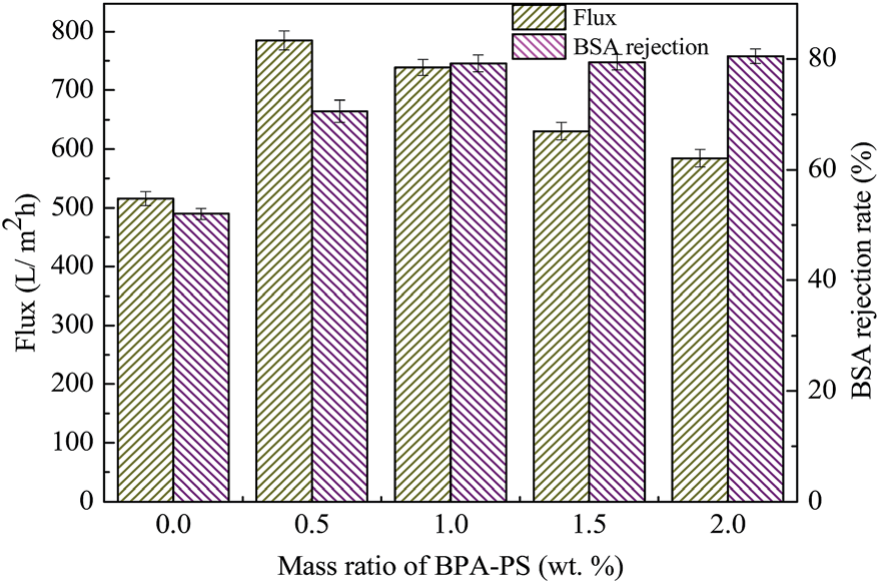
The pure water flux and BSA rejection rate. Coagulation water bath temperature was 323 K.

**Fig. 11:**
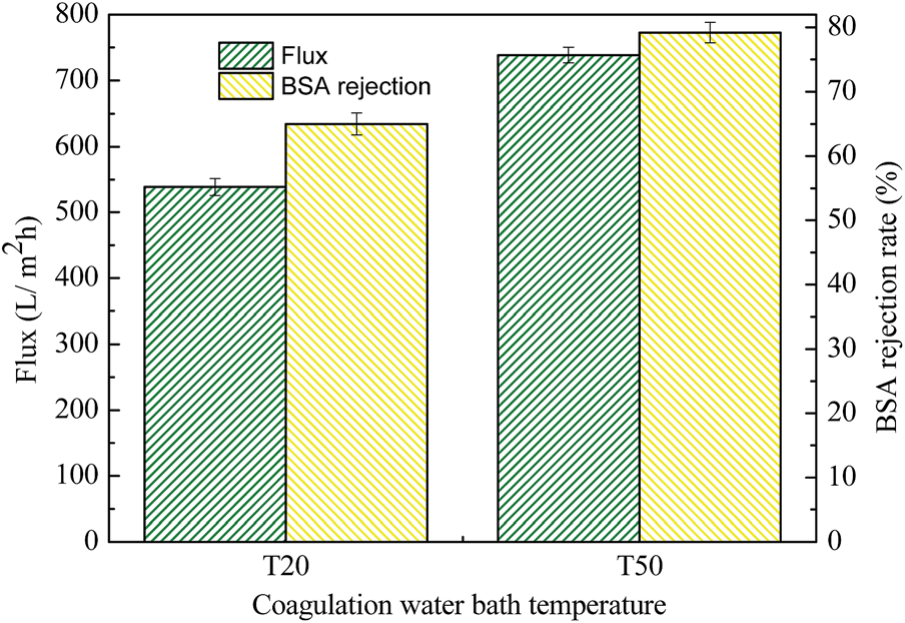
The pure water flux and BSA rejection rate of MBPA-PS-1-20 and MBPA-PS-1-50. Coagulation water bath temperature was 323 K.

The effect of BPA-PS and different temperatures of coagulation water bath on pure water flux and BSA rejection rate were displayed in Fig. 10 and 11, respectively. The pure water flux in Fig. 10, which the coagulation water bath temperature was set at 323 K, increased first and then decreased with BPA-PS. The increasing trend of pure water flux was attributed to the hydrophilicity group in BPA-PS while the decreasing trend was due to the increased viscosity by BPA-PS. These data were consistent with the mean pore size (*r*_m_) listed in Table 4 and water contact angle in Fig. 9. The optimum pure water flux (739 L m^−2^ h^−1^) and BSA rejection (79.2%) were obtained from the membrane with 1.0 wt% BPA-PS.

T20, T50, as shown in Fig. 11, were the coagulation water bath temperature. The phase separation process underwent NIPS method in T20 while the phase separation followed by RTIPS process in T50. It was evident that the pure water flux and BSA rejection rate of MBPA PS-1-50 which prepared by RTIPS method were higher than that of MBPA PS-1-20 by NIPS process. This phenomenon unanimous with that the dense skin top surface and the finger-like cross-section were obtained *via* NIPS while the porous top surface and the sponge-like cross-section were acquired by RTIPS. Porous top surface and sponge-like structure were conducive to high pure water flux and BSA rejection rate, respectively. This phenomenon was a convincing evidence for that RTIPS mechanism had the potential for high pure water flux and BSA rejection rate.

### 3.10 Antifouling property of PES/BPA-PS hollow fiber membrane

**Fig. 12:**
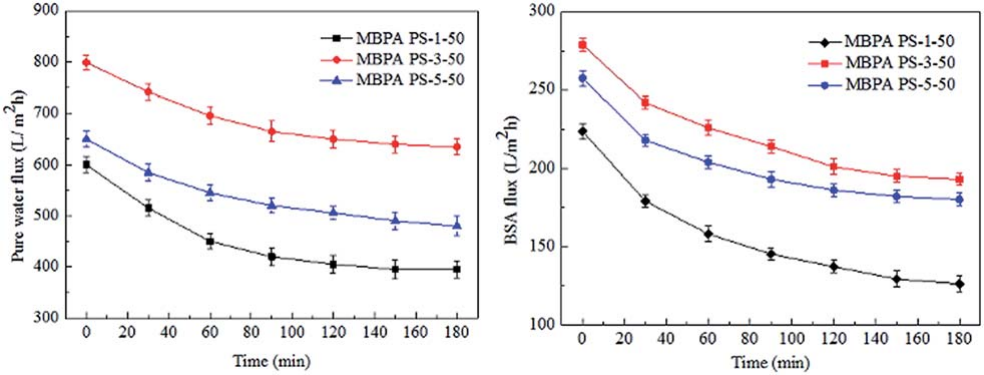
The change of pure water flux and BSA flux with time.

**Fig. 13:**
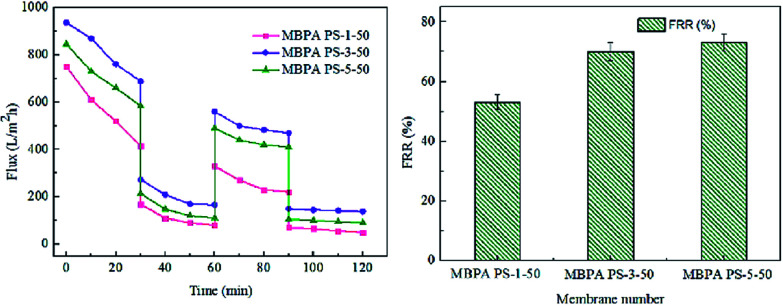
The recycles and FRR of the membranes.

The FRR, pure water flux and BSA flux with time of the membranes were shown in Fig. 12 and 13, respectively. For the membranes prepared by RTIPS process, the FRR of the membranes with BPA-PS (MBPA PS-3-50, MBPA PS-5-50) was higher than the pure PES membrane (MBPA PS-1-50). This phenomenon was coincident with the test results of water contact angle and AFM. As for the changes of pure water flux and BSA flux with time, it decreased in the initial stage then changed little with time. The pure water flux and BSA flux with time of the MBPA PS-1-50, as illustrated in Fig. 12 and 13, displayed a faster descend trend than the MBPA PS-3-50 and MBPA PS-5-50. These changes illustrated the better antifouling performance of the membranes with BPA-PS.

### 3.11 Comparison with other membranes

As listed in Table 6, the properties of the membranes in this paper were compared with other membranes in the literature. Both the high water flux and BSA rejection could be acquired *via* RTIPS (Fig. 10 and 11). Furthermore, the anti-fouling properties were improved by the addition of BPA-PS.

**Table 6:** The comparison with other membranes

Membranes	Preparation method	Water bath temperature (K)	Flux (L m^−2^ h^−1^)	Re (%)	Reference
PES/DMAC/DEG	VIPS	313	1090 ± 35	10 ± 0.3 (BSA, 67 000)	41
PES/Mg(OH)_2_/DMAC	NIPS	298	700 ± 45	90 ± 2.7 (BSA, 67 000)	43
PVDF/WA/DMAC	NIPS	298	70.6 ± 5	81 ± 2.1 (BSA, 67 000)	44
PES/PDMAEMA-*b*-PES-*b*-PDMAEMA	NIPS	298	145 ± 9	70 ± 2.1 (BSA,67 000)	45
PES/P(H–P–A)/DMAC	NIPS	298	137 ± 10	57 ± 0.9 (BSA, 67 000)	46
PES/DMAC/PEG200	RTIPS	313	1040 ± 56	39 ± 3.1 (BSA, 67 000)	18
PSF/HBPE/DMAC/PEG400	RTIPS	343	375 ± 17	≥90 ± 3 (DEX,1440 kDa)	20
PES/TiO_2_/DMAC/DEG	RTIPS	333	1046 ± 45	75 ± 2 (BSA, 67 000)	21
PES/BPA-PS/DMAC/DEG	RTIPS	323	738.5 ± 25	79 ± 2.1 (BSA, 67 000)	This study

## 4 Conclusion

This paper presented that a novel polyethersulfone (PES) hollow fiber membrane was modified by the addition of bisphenol sulfuric acid (BPA-PS) which was fabricated *via* reverse thermally induced phase separation (RTIPS) process. Bisphenol sulfuric acid (BPA-PS) was synthesized by click chemistry and was blended to improve the hydrophilicity of PES hollow fiber membranes. The influences of different ratios of BPA-PS and cloud point on the PES membrane properties (including penetration performance, membrane morphology and anti-fouling property *etc.*) were investigated. The cloud point, which was used to explore the temperature of the phase separation, changed a little bit with the addition of BPA-PS. Nevertheless, the water contact angle displayed the decreasing trend from 92.6° to 61.6° with the addition of BPA-PS. The flux of pure water (*J*_w_) and BSA rejection rate (*R*) of the membranes increased first and then decreased with the increased BPA-PS. And the optimal pure water flux (739 L m^−2^ h^−1^) and BSA rejection rate (79.2%) were obtained from the membrane with 1 wt% BPA-PS.

With regarded to the influence of coagulation water bath temperature on membrane performance, the mechanism of membrane formation transformed NIPS to RTIPS when the temperature of coagulation water bath increased to the cloud point. In the meantime, the finger-like cross sections changed into sponge-like structures. As for the membranes prepared by RTIPS, the pure water flux and BSA rejection rate were both higher than the membranes achieved from NIPS. This phenomenon confirmed that the membrane produced by RTIPS had better performance than that *via* NIPS process.

Generally speaking, the PES/BPA-PS membranes, which prepared by reverse thermally induced phase separation (RTIPS), displayed excellent properties on pure water flux, BSA rejection rate and anti-pollution performance *etc.* This research offered a novel approach to combine reverse thermally induced phase separation (RTIPS) and click chemistry on the PES/BPA-PS hollow fiber membrane preparation.

## Conflicts of interest

There are no conflicts to declare.

## Supplementary Material
